# The Impact of VEGF-C-Induced Dural Lymphatic Vessel Growth on Ischemic Stroke Pathology

**DOI:** 10.1007/s12975-024-01262-9

**Published:** 2024-06-01

**Authors:** Meike Hedwig Keuters, Salli Antila, Riikka Immonen, Lidiia Plotnikova, Sara Wojciechowski, Sarka Lehtonen, Kari Alitalo, Jari Koistinaho, Hiramani Dhungana

**Affiliations:** 1 Neuroscience Center, Helsinki Institute of Life Science (HiLIFE), University of Helsinki, 00014 Helsinki, Finland; 2 A.I. Virtanen Institute for Molecular Sciences, University of Eastern Finland, 70210 Kuopio, Finland; 3 Wihuri Research Institute and Translational Cancer Medicine Program, University of Helsinki, 00014 Helsinki, Finland; 4 Drug Research Program, Division of Pharmacology and Pharmacotherapy, University of Helsinki, 00014 Helsinki, Finland

**Keywords:** VEGF-C, Ischemic stroke, Dura mater, Lymphatic vessels, Lymph

## Abstract

Timely relief of edema and clearance of waste products, as well as promotion of anti-inflammatory immune responses, reduce ischemic stroke pathology, and attenuate harmful long-term effects post-stroke. The discovery of an extensive and functional lymphatic vessel system in the outermost meningeal layer, dura mater, has opened up new possibilities to facilitate post-stroke recovery by inducing dural lymphatic vessel (dLV) growth via a single injection of a vector encoding vascular endothelial growth factor C (VEGF-C). In the present study, we aimed to improve post-stroke outcomes by inducing dLV growth in mice. We injected mice with a single intracerebroventricular dose of adeno-associated viral particles encoding VEGF-C before subjecting them to transient middle cerebral artery occlusion (tMCAo). Behavioral testing, Gadolinium (Gd) contrast agent-enhanced magnetic resonance imaging (MRI), and immunohistochemical analysis were performed to define the impact of VEGF-C on the post-stroke outcome. VEGF-C improved stroke-induced behavioral deficits, such as gait disturbances and neurological deficits, ameliorated post-stroke inflammation, and enhanced an alternative glial immune response. Importantly, VEGF-C treatment increased the drainage of brain interstitial fluid (ISF) and cerebrospinal fluid (CSF), as shown by Gd-enhanced MRI. These outcomes were closely associated with an increase in the growth of dLVs around the region where we observed increased *vefgc* mRNA expression within the brain, including the olfactory bulb, cortex, and cerebellum. Strikingly, VEGF-C-treated ischemic mice exhibited a faster and stronger Gd-signal accumulation in ischemic core area and an enhanced fluid outflow via the cribriform plate. In conclusion, the VEGF-C-induced dLV growth improved the overall outcome post-stroke, indicating that VEGF-C has potential to be included in the treatment strategies of post-ischemic stroke. However, to maximize the therapeutic potential of VEGF-C treatment, further studies on the impact of an enhanced dural lymphatic system at clinically relevant time points are essential.

**Supplementary Information:**

The online version contains supplementary material available at 10.1007/s12975-024-01262-9.

## Introduction

Ischemic stroke results in death of brain cells leading to sensorimotor and cognitive impairments. Sudden and transient reduction of blood flow can cause ionic imbalance, acidosis, excitotoxicity, oxidative stress, blood–brain barrier (BBB) disruption, glial cell activation, and leukocyte infiltration into the affected area; these contribute to the ischemic brain injury [1]. Edematous and necrotic cell death during the early phase after stroke is particularly detrimental and considered a major risk factor for morbidity and mortality in stroke patients [2]. Ischemic brain damage is aggravated by further infiltration of peripheral leukocytes and secretion of inflammatory cytokines at later time points [3]. Furthermore, the circulation and drainage of brain interstitial fluid (ISF) and cerebrospinal fluid (CSF) are significantly reduced in the acute phase post-stroke, impairing waste clearance, an essential process for brain homeostasis, which is required for the proper and healthy functioning of cerebrovascular and parenchymal brain cells [4, 5]. Therefore, treatment strategies aimed at improving circulation and CSF drainage are expected to improve behavioral outcome post-stroke.

The presence of a lymphatic vessel (LV) network in the dura mater, the outermost meningeal layer surrounding the central nervous system (CNS), was recently described by two independent research groups [6, 7]. Vascular endothelial growth factor C (VEGF-C), a key regulator of lymphangiogenesis, was shown to drive the postnatal development of dural lymphatic vessels (dLVs) and VEGF-C delivery could be used for their further expansion in adults [8]. The dLVs were shown to drain macromolecular tracers administered into ISF and CSF into the extracranial lymphatic vessels leading to cervical lymph nodes (cLNs) [6, 7, 9]. Deep cervical LNs (dcLNs) are essential in the circulation of immune cells between the CSF and peripheral immune system, which contribute to brain immune surveillance [10, 11]. Following the discovery of dLVs, several groups have explored the possibility of modifying their function in various neurodegenerative and neuroinflammatory diseases and brain cancer and metastasis [12–16].

VEGF-C promotes lymphatic endothelial cell migration, proliferation, and formation of de novo LVs through binding to its high-affinity receptor VEGFR3 [8]. The importance of VEGF-C has been demonstrated by a study showing that embryos lacking both Vegfc alleles (Vegfc^−/−^ mice) are not viable [17]. The significance of VEGF-C and VEGFR3 signaling for dLV function in adult mice has been demonstrated by induction of dLV atrophy after deletion of VEGF-C or VEGFR3, or by trapping the VEGFR3 ligands VEGF-C and VEGF-D by transgenic or viral vector mediated expression of a soluble VEGFR3 [6, 8]. Both genetic and AAV-induced atrophy of dLVs inhibited ISF/CSF drainage into cervical LNs [6, 8, 9]. However, mice born without a functional dLV system showed a normal brain water content and intracranial pressure, suggesting that the developmental defect in ISF/CSF clearance is compensated by other mechanisms [6]. The overexpression of VEGF-C, on the other hand, has been reported to improve both brain perfusion and ISF/CSF drainage into dorsal cervical LNs at least in aged mice [12]. Interestingly, several studies have shown that VEGF-C has also neurotrophic effects and can promote neurogenesis [18–21]. Moreover, ectopic expression of VEGF-C has been documented to provide T cell-mediated immune response against glioblastoma [22].

As lymphatic vessels function in the clearance of fluid, solutes, and waste from the CNS [6, 23, 24], we considered that stimulation of lymphangiogenesis through overexpression of VEGF-C may have beneficial effects in acute brain insults associated with cerebral edema and waste accumulation. We also considered that the anti-inflammatory and neurogenic effects of VEGF-C could provide additional benefits during neuroinflammation. Thus, we tested if VEGF-C-induced lymphangiogenesis can increase ISF and CSF circulation after stroke and thereby provide neuroprotection, improving behavioral outcomes after ischemic stroke.

## Materials and Methods

### Ethical Approval

The National Animal Experiment Board of Finland approved all animal experiments in accordance with the Council of Europe Legislation and Regulation for Animal Protection. All experiments followed national guidelines for the usage and welfare of laboratory animals.

### Animals and Experimental Design

Young adult male mice, aged 6–8 weeks, were purchased from two different vendors, either Envigo (C57BL/6JOlaHsd) or Janvier (C57BL/6JRj), and housed in groups under controlled temperature (22 ± 2 °C), with a 12/12 h light/dark cycle and access to food and water ad libitum. We chose to use C57BL strains since they are the most commonly used and the best-characterized mice when using a transient middle cerebral artery occlusion (tMCAo) as a stroke model. As listed in Table 1, the mice were divided into four cohorts. The mice underwent a single intracerebroventricular (i.c.v.) injection of 1 × 10^10^ viral particles of serotype 9 adeno-associated viral (AAV9) vector encoding full-length mouse VEGF-C (AAV9-mVEGF-C-FL) or an AAV without payload (Ctrl) 14 days before sampling for qPCR analysis, ischemic surgery, sham surgery, respectively. Mice of the third cohort were subjected to a single injection of 5.36 × 10^10^ viral particles of either VEGF-C or Ctrl 35 days before ischemia. Mice assigned to qPCR analysis were randomized to either VEGF-C or Ctrl treatment. Mice in other cohorts were randomized into the following four treatment groups: (1) VEGF-C-injection plus tMCAo surgery (tMCAo;VEGF-C group, C57BL/6JOlaHsd mice) or (2) VEGF-C-injection plus sham surgery (sham;VEGF-C, C57BL/6JOlaHsd mice) and (3) Ctrl-injection plus tMCAo surgery (tMCAo;Ctrl, C57BL/6JRj mice) or (4) Ctrl-injection plus sham surgery (sham;Ctrl, C57BL/6JRj mice). To exclude the possibility that the outcome of stroke is surgeon-dependent, the surgery was performed by two persons, cohort 1 and 2 surgeries by MHK and cohort 3 and 4 by HD. All randomizations were carried out using GraphPad QuickCalcs (GraphPad software, CA, USA).

**Table 1:** Overview of the cohorts and treatment regimens

Cohort	tMCAO;VC	Excluded	sham;VC	Excluded	tMCAO;Ctrl	Excluded	sham;Ctrl	Excluded	Virus-injection/remark
1			6	0			6	0	− 14 days prior sampling
2	(21) 15	7	(12) 11	1	(20) 17	3	(11) 11	0	− 14 days prior tMCAO/sham surgery
3	(20) 17	3	(8) 7	1	(20) 15	5	8	0	− 35 days prior tMCAO/sham surgery
4	(9) 7	2	(9) 6	3	(9) 6	3	(9) 6	3	− 14 days/Gd-enhanced MRI
Sum	50	12	35	5	49	11	34	3	

Detailed summary of mice’s treatment regimens, group sizes, and numbers of excluded animals per group and treatment. Altogether, 18.5% of the mice were excluded according to pre-set criteria. VC = adeno-associated virus encoding mouse VEGF-C-FL; Ctrl = AAV without payload; (n) = mouse numbers (in brackets) originally assigned to the treatment groups; n = mouse numbers after exclusion; Gd = gadolinium

### Intracerebroventricular Injection

For the i.c.v. injection, the mice were anesthetized using 5% isoflurane in 30% O_2_ and 70% N_2_O and placed into the stereotactic frame (David Kopf Instruments, CA, USA). The anesthetic was reduced to 2% for maintenance and the body temperature was maintained at 36.5 ± 0.5 °C using a homoeothermic blanket (PanLab, Barcelona, Spain). The head of the anesthetized mouse was fixed using the stereotactic frame. After making an incision on the shaved and disinfected scalp, a blunt needle (33G) of a 5-µl Hamilton syringe (Hamilton, NV, USA) was aligned to the following coordinates: + 0.3 mm anterior/posterior (A/P), + 1.0 medial/lateral (M/L) from bregma. Using a dental burr head (Meissinger, Duesseldorf, Germany), a small hole was drilled into the skull above the injection site without injuring the meninges. Subsequently, the needle was inserted into the brain tissue (− 2.0 mm dorsal/ventral (D/V) below the dural surface). A single dose of either VEGF-C or Ctrl diluted in phosphate-buffered saline (PBS, final volume of 4 µl) was infused into the left ventricle with a microinfusion pump (Harvard Apparatus, MA, USA) at a speed of 0.5 µl/min for 8 min. After injection, the needle was kept at the injection site for 4 min before slow withdrawal.

### Tissue Samples for RNA Expression Study

To determine the effect of VEGF-C administration on overall gene expression of VEGF-C in different brain areas at the time of ischemic stroke, mice were injected with VEGF-C or Ctrl 14 days before sampling without stroke surgery. Samples from the cerebellum, cortex, hippocampus, olfactory bulb, and striatum were dissected, snap-frozen, and stored at – 70 ℃ until further processing. For total cellular RNA extraction, RNeasy Mini Kit (QIAGEN, Hilden, Germany) was used according to the manufacturer’s instructions, and the concentration and purity of the samples were determined using a NANODrop 1000 (Thermo Fisher). Complementary DNA (cDNA) was synthesized from 500 ng of total RNA using random hexamer primers as a template and Maxima reverse transcriptase (Life Technologies, CA, USA). Following the cDNA synthesis (2.5 ng/µl final concentration), qPCR was run using the StepOne Plus Real-time PCR system according to manufacturer’s instructions (Applied bioscience, CA, USA) with the following TaqMan assay-on-demand target mixes: *vegfc* (Mm00437310_m1), *aqp4* (Mm00802131_m1), and *b2m* (Mm 00437762_m1). The expression levels were obtained by normalization to *b2m* and are presented as relative expressions.

### Transient Middle Cerebral Artery Occlusion

The tMCAo surgery was adapted from Kolosowska et al. [25]. Briefly, mice were anesthetized, and a neckline incision was made. A silicon-coated 6–0 nylon monofilament (0.21 ± 0.02 mm in diameter, Doccol, MA, USA) was introduced into the left or right (only mice assigned to the Gd-contrast agent MRI experiments) external carotid artery and advanced through the internal carotid artery to block the middle cerebral artery (MCA) approximately 10 mm after its bifurcation to the internal and external carotid arteries. Following 45 min of occlusion, which typically leads to infarction in the striatum and overlaying cortex, the blood flow was restored by withdrawing the filament. Trans-cranial laser Doppler flowmetry (LDF) was used to confirm stable MCA occlusion. Therefore, a fiber-optic probe (Moor Instruments, UK) was mounted to the intact skull bone in the MCA territory to confirm appropriate blood flow reduction followed by proper restoration. Sham mice underwent the same surgical procedure except the filament was withdrawn immediately after introducing it halfway to the internal carotid artery. For analgesia, buprenorphine (0.03 mg/kg) was injected shortly before and 24 h after surgery. The mice were allowed to recover in a recovery chamber for 60 min and returned to their home cages, which were kept on heating pads for 24 h.

### Evaluation of the Infarction and Edema Volumes

Magnetic resonance imaging (MRI) was carried out on anesthetized (isoflurane anesthesia as described before) ischemic mice to determine lesion and edema volumes. As done previously, the imaging was performed at three days post-ischemia (dpi), a time point at which the stroke and edema volumes have fully expanded [25, 26]. While stroke volumes could be quantified from 24 h post-ischemia onwards, early cytotoxic edema, which does not yet cause an overall tissue expansion, transforms over the first days towards vasogenic, tissue-expanding edema [27]. Thus, quantification is most reliable for stroke and edema volumes at three dpi. MRI was performed on a vertical 9.4 T Oxford NMR 400 magnet. Using a quadrature volume coil for transmission and reception, we obtained multi-slice T2-weighted images (repetition time (TR) 3000 ms, echo time (TE) 40 ms, Matrix size 128*256, field of view 19.2 mm, slice thickness 0.8 mm and slices 12) with double spin-echo sequence with adiabatic refocusing pulse. The total infarction volume, the volume of the left intact hemisphere and the volume of the right intact hemisphere were calculated from 12 consecutive slices per animal. The MRI images were analyzed using an in-house aedes software under MATLAB environment (Math-Works, MA, USA). The relative percentages of infarction volume and edema were calculated following Shuaib et al. [28]: Infarct size = [volume of the left hemisphere – (volume of the right hemisphere – measured infarction volume)] / volume of the left hemisphere:

Edema = (volume of right hemisphere − volume of left hemisphere) / volume of left hemisphere.

All analyses were performed blinded to the study groups.

### Behavioral Testing

#### Catwalk Gait Analysis

The CatWalk automated gait analysis system (Noldus Information Technology, Wageningen, Netherlands) is commonly used to assess gait and locomotor parameters following ischemia and was adapted according to previous studies [25, 29, 30]. The animal training and testing sessions were performed in a dimmed room (< 20 lx of illumination). For the assessment, an enclosed glass walkway (9 × 60 cm) was illuminated with a green, internally reflected light. A high-speed camera was placed 40 cm below the glass walkway to capture the green light reflected by the paws upon glass contact and transformed it into a digital image. The intensity threshold was set to 0.11/ 0.12 (average to match mice of differing weights) and the camera gain was set to 18; the maximum allowed speed variation was set to 50%. For a successful run, each mouse had to walk spontaneously at its own speed without interruption. A minimum of three uninterrupted runs per mouse was saved. The animals underwent two training sessions, one under normal light and one in dimmed light. Baseline (BL) recording was performed on the day before ischemic stroke surgery. All mice (i.c.v. VEGF-C/ Ctrl -14 dpi) underwent CatWalk testing at seven dpi blinded to the treatment groups.

#### Neurological Score

General and focal neurological deficits were assessed at three and seven dpi as described elsewhere [31]. The general dysfunction was scored based on the following subjects: fur (0–2), ears (0–2), eyes (0–4), posture (0–4), spontaneous activity (0–4), and epileptic status (0–12). Likewise, focal deficits were assessed on the following parameters: body asymmetry (0–4), gait (0–4), climbing on a 45°-inclined surface (0–4), circling behavior (0–4), front limb symmetry (0–4), and whisker response to light touch (0–4). The sum of general and focal deficits was calculated and scored from zero (no deficits) to 56 (poorest neurological outcome in all categories) blinded to the study groups.

#### Latency to Move Test

The locomotor activity was evaluated at 3 dpi by the latency to move test as described previously [32]. In brief, the animals (i.c.v. VEGF-C/ Ctrl at − 14 dpi) were placed on a table and the time to move one body length (about 7 cm) was recorded. Each mouse was tested three times and the mean value was calculated blinded to the study groups.

#### Corner Test

The corner test, designed to detect unilateral aberrations of sensory and motor functions, was performed in ischemic mice, treated 5 weeks prior tMCAo surgery with VEGF-C or Ctrl. For the test two opaque boards (approximately 30 cm × 20 cm × 1 cm) were connected in a 30° angle [33]. At the corner, a small opening was left to motivate mice, which were placed halfway into the corner, to enter deeper into the corner. As the mouse walks towards the opening the vibrissae of both sides are stimulated. The naïve mouse should rear and turn equally often toward either the left or right corner, while ischemic mice typically rear and turn towards the non-injured (ipsilateral) side. Ten trials were counted per mouse (turns without rearing did not count). The test was performed at three dpi blinded to the study groups.

### Gadolinium Contrast-Enhanced Dynamic Imaging

Fourteen days after a single i.c.v. injection of VEGF-C or Ctrl, mice underwent tMCAo or sham surgery. After 24 h, mice were subjected to gadolinium (Gd) contrast-enhanced dynamic MRI measurements. In brief, mice were anesthetized with an intraperitoneal injection of Ketamine (75 mg/kg) and Domitor (1 mg/kg, Pfizer, Helsinki, Finland) and each mouse received a single dose of gadolinium contrast agent (Omniscan (0.5 mol/L solution), GE Healthcare; IL, USA) i.c.v. using the same coordinates as for viral particle injections. Each animal was injected with 2 µl Gd at 1:16 dilution in artificial cerebrospinal fluid [34], at a speed of 0.5 µl/min, and the needle was kept in place for 4 min. After needle retraction, the mouse was immediately secured to an animal holder using ear bars and a bite bar and moved into the MRI scanner. The Gd-tracer dynamics were followed at a horizontal 7 T magnet with linear volume transmit coil and quadrature surface receiver coil (Bruker Pharmascan, ParaVision 5.1 system, Germany). Dynamic Gd-contrast enhanced MRI scans were acquired with a 3 min temporal resolution for 100–120 min, using a T1-weighted steady state gradient echo (Fast Imaging with Steady-state Precession, FISP) 3D sequence (TR/TE/flip 12 ms/4 ms/15° 130 µm^3^ isotropic, scan time per image 3 min) (Yasmin et al., 2019). Interleaved, ~ 45 min post-Gd injection, a multi-echo gradient echo anatomical scan (TR 72 ms, averaged over 13 echo images, first TE 3.02 ms, echo spacing 3.63 ms, flip 16°, 130 µm^3^ isotropic) was obtained as a single 15-min lasting scan. The body temperature (36.5 ± 0.5 °C) and respiratory rate (60–70 bpm) were controlled using a water-heated blanket and isoflurane anesthesia within the magnet.

For analysis, the series of fast T1-weighted images (whole brain 3D FISP every 3 min over 100 min) were aligned to a 4D-data set. Pre-processing included movement corrections using Advanced Normalization Tools (ANTS—Advanced Normalization ToolS (RRID:SCR_004757); http://www.picsl.upenn.edu/ANTS/), followed by smoothing at a rate of sigma 0.1 with an in house-written macro under Matlab environment (v2017b, MathWorks). Regions of interest (ROIs) were manually drawn into the following areas: infarcted cortex (Additional file 1. Fig. S1 a.1); peri-ischemic cortex (rostral of lateral ventricle and ipsilateral to infarct area; (Additional file 1. Fig. S1 a.2); and outflow area at the basal brain (ventrally from rhombencephalon and pons, red ROI, Additional file 1. Fig. S1 a.3). Infarcted and peri-infracted areas were distinguishable by the MGRE-anatomical scan. MR images and the Allen mouse brain atlas were used to choose the outflow area from the basal brain (https://mouse.brain-map.org). Gd-induced signal intensity changes over time were assessed for each ROI. In addition, time-to-peak-enhancement maps were created using an in-house-written macro under a Matlab environment. These maps show the absolute time needed to reach the maximum Gd-enhanced intensity for each voxel, min post-Gd-injection as units. For analyses, time-to-peak-enhancement maps were overlaid with the MGRE-anatomical scans (movement corrected), and ROIs were drawn for relevant areas as follows: the ischemic cortex of stroked mice or the corresponding area in sham mice (Additional file 1. Fig. S1 b.1), the olfactory bulb (Additional file 1. Fig. S1 b.2), which is a main parenchymal outflow area, and the cribriform plate, through which CSF is drained to lymphatic vessels in the nasal mucosa towards cervical lymph nodes [5].

### Tissue preparation

After the last MRI and behavioral tests, the mice were terminally anesthetized using 250 mg/kg Avertin (2,2,2-tribromoethanol in tertiary amyl alcohol) and perfused transcardially with ice-cold, heparinized (2500 IU/L, Leo Pharma A/S, Ballerup, Denmark) saline. The head was dissected as previously described [8]. Briefly, a fine pair of scissors was inserted through the cisterna magna. After cutting along the parietal bone lines on both sides, the parietal part was carefully removed without harming the dura mater. Next, the brain was removed carefully from the skull bone before the spinal canal was opened up by incisions on the lateral sides of the canal. The remaining tissue parts were removed with extra care not to harm the dura mater attached to the basal cranium. All dissected tissues were immediately immersed in ice-cold 4% paraformaldehyde (PFA) for 22–24 h at 4 °C, followed by washes in PBS. Next, brains were transferred to 30% sucrose for 48 h for cryopreservation and snap-frozen in liquid nitrogen before being stored at – 70 °C. The dural samples were stored at 4 °C in PBS containing 0.04% CiNaH_3_ until further processing.

### Immunohistochemical Staining

Six randomly picked brains of mice injected with VEGF-C or Ctrl at − 14 dpi were sectioned with a cryostat (Leica Microsystems, Wetzlar, Germany) into 20-µm-thick Sects. (400 µm apart). Six to eight sections were blocked in 10% normal goat serum and incubated overnight at room temperature (RT) with ionized calcium-binding adapter molecule-1 (Iba1, 1:250, Wako Chemicals, Tokyo, Japan), arginase-1 (Arg1, 1:200, Santa Cruz Biotechnology, TX, USA), glial fibrillary acid protein (GFAP, 1:500, Dako, Glostrup, Denmark), and doublecortin (DCX, Cell Signaling Technology, MA, USA) antibodies. Next, the sections were washed and incubated in secondary antibodies conjugated to Alexa Fluor 568 (1:200, ThermoFisher Scientific, Waltham, USA). Negative controls for each staining were prepared in parallel by incubating sections overnight without primary antibodies. The brain sections were imaged at 4 × magnification using a Zeiss Axio Observer (Carl Zeiss AG, Jena, Germany) and stitched together using the ZEN blue software (Zeiss). For Iba-1 and GFAP staining, two different regions of interest (ROI) were taken from the peri-ischemic area and quantified using ImagePro Plus software (Media Cybernetics, MD, USA). To quantify immunoreactivity, a predefined intensity range was chosen to minimize the background artifacts, and the mean percentage of the immunoreactive area was calculated (Fig. 6b, d). Accordingly, a predefined area of DCX-stained brain sections was quantified at 10 × magnification (Fig. 6h). For the quantification of Arg1-positive immunoreactivity, ROIs were drawn into the ischemic area at the ventral border area (Fig. 6f). All analyses were done blinded to the study group.

### Whole-Mount Staining and Analysis of Dura Mater Lymphatics and Vasculature

For whole-mount staining of the PFA-fixed dura mater, the tissue was initially permeabilized in 0.3% Triton X-100 in PBS (PBS-TX) at RT, followed by 1–2 h-blocking of the tissues with 5% donkey serum/2% bovine serum albumin/0.3% PBS-TX (DIM). Next, the durae matres were incubated overnight at 4 °C with the following primary antibodies: goat anti–mouse podocalyxin (1:500, AF1556; R&D Systems), rat anti-mouse lymphatic vessel endothelial hyaluronan receptor 1 (LYVE-1, 1:300, MAB2125; R&D Systems), or polyclonal rabbit anti-mouse LYVE-1 (1:1000, (Karkkainen et al., 2004)) diluted in DIM. The samples were then washed at RT with PBS-TX and incubated with the secondary antibodies (Alexa 488 donkey anti-goat, 1:500, Alexa 594 donkey anti-rat, 1:500, and Alexa 594 donkey anti-rabbit, 1:500) in PBS-TX at 4 °C overnight. Finally, the samples were post-fixed for 5 min in 1% PFA, washed with PBS at RT, and stored in PBS containing 0.05% sodium aside (Na_2_N3) at 4 °C until imaging. LYVE-1-positive dLVs both in dorsal and basal cranium were imaged using an Axio Zoom.V16 fluorescence stereo zoom microscope (Zeiss) equipped with an ORCA-Flash 4.0 digital CMOS camera (Hamamatsu Photonics; Japan). The dorsal areas shown in figures include the surroundings of the superior sagittal sinus (SSS), the confluence of the sinuses (COS), and transverse sinuses (TS). The basal areas shown in the figures include the surroundings of cranial nerves II (optic nerve, CNII), pterygopalatine/middle meningeal artery (PPA/MMA), and the cervical vertebra surroundings (Fig. 7c). Podocalyxin-positive dural blood vessels (dBVs) were imaged in the dorsal skull (Fig. 7c). Quantification of dLVs and dBVs was done by calculating the LYVE-1 and podocalyxin positive area percentage of the total imaged area, respectively. Image acquisition and quantitative analysis were done using the ZEN 2012 software (Zeiss) and Fiji software, respectively. Quantification of dBVs was done from the same ROI at both sides of SSS directly with the thresholding tool of ImageJ software. Brightness and contrast for publication images were adjusted using ImageJ software.

### Statistical Analysis and Exclusion Criteria

Statistical analyses were carried out using GraphPad Prism software (GraphPad Software, Inc., CA, USA) by running the one-way ANOVA followed by Bonferroni’s, Dunn’s, or Tukey’s multiple comparison tests, the two-way ANOVA with Tukey’s multiple comparison test, or, whenever appropriate, the two-tailed Student’s unpaired *t* test. Exclusion criteria were set before the start of the experiments. Altogether, 85 (31.8%) out of the 278 mice were excluded from the experimental sets due to insufficient ischemic occlusion as determined by LDF (< 70% decrease of blood flow in the MCA territory), by hemorrhagic transformation, excessive body weight loss (> 20%), sudden death, or inappropriate injection of the Gd-contrast agent (for details see Table 1). All the studies were carried out blinded to the treatment groups and *p* ≤ 0.05 was considered statistically significant. The data are presented as mean ± standard error of the mean (SEM).

## Results

### AAV-Mediated Transduction and Expression of VEGF-C in the Mouse Brain

**Fig. 1:**
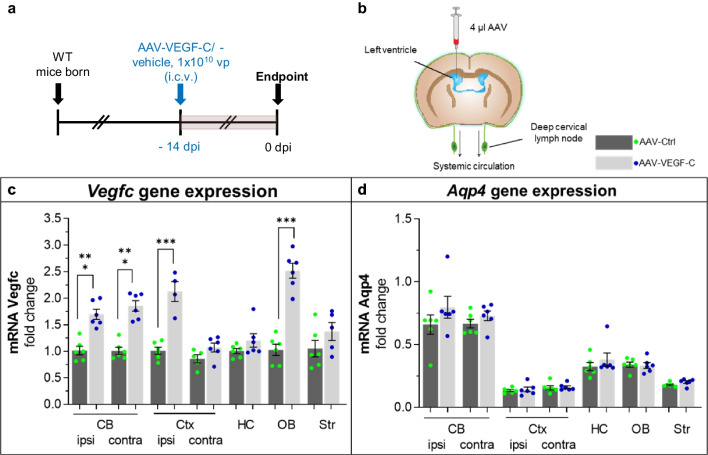
AAV-VEGF-C transduction increase in *Vegfc* mRNA in several brain regions. **a** Experimental outline showing time points for administration and sample taking. **b** A schematic illustration of the AAV injection site 14 days before sample taking. **c** *Vegfc* and **d** *Aqp4* gene expression levels in cortex, cerebellum (ipsi- and contralateral to inj.), hippocampus, olfactory bulb, and striatum 2 weeks after Ctrl and VEGF- C injection. *n* = 5–6/per group; data points represent individual mice. *P* values were calculated with ordinary one-way ANOVA with Tukey’s post hoc multiple comparison test. Data are expressed as mean ± SEM, ***p < 0.001. *CB* cerebellum, *Ctx* cortex; *HC* hippocampus; *OB* olfactory bulb; *Str* striatum

We used a single i.c.v. injection of 1 × 10^10^ AAV-VEGF-C particles per mouse as it has been shown to induce significant lymphangiogenesis in dorsal and basal dLVs as reported previously [8]. We verified VEGF-C expression in different brain areas after similar VEGF-C injection (Fig. 1a, b). Quantitative real-time PCR (qPCR) revealed a significant increase of *Vegfc* transcripts in RNA isolated from various brain regions two weeks after the AAV-VEGF-C injection *vs* AAV-Ctrl (without payload). The increase in VEGF-C vs. Ctrl mice was ≥ twofold in the cerebellum and olfactory bulb (*p* < 0.001), and ~ 1.5-fold in the cortex (*p* < 0.01) (Fig. 1c). In contrast, the expression of *Aquaporin4* (*Aqp4*) mRNA, which encodes a water channel protein expressed in astrocyte endfeet at the interface between brain and blood circulation [35], was not altered in VEGF-C vs. Ctrl mice (Fig. 1d). These data suggest that i.c.v delivery of VEGF-C induces increased *Vegfc* expression in the brain without affecting *Aqp4* expression.

### Overexpression of VEGF-C Does Not Affect Ischemia-Induced Brain Infarct Volume or Edema

**Fig. 2:**
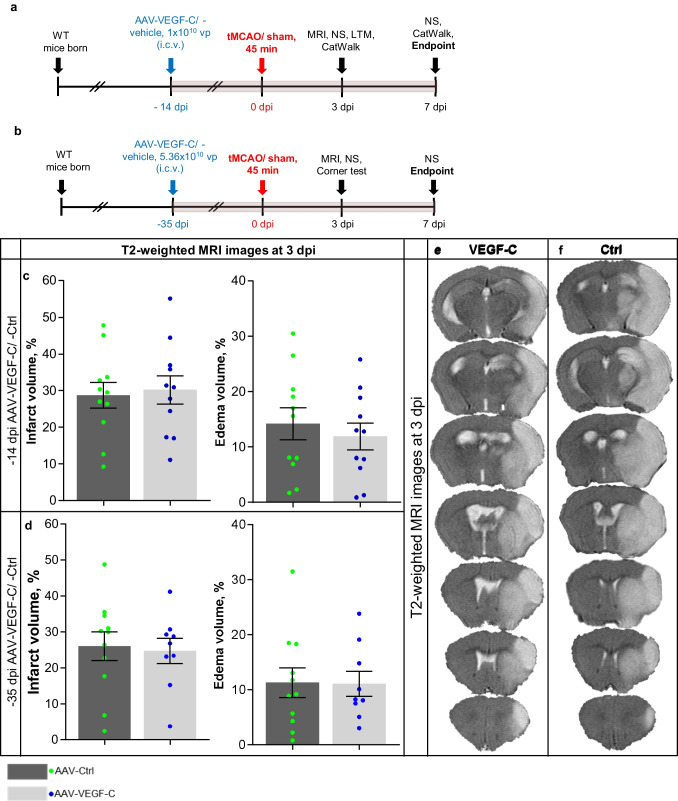
Infarct and edema volumes are not altered by VEGF-C overexpression. **a**, **b** Experimental outlines showing time points for administration, ischemic surgery, behavioral testing, and sample taking. **c**, **d** Infarct and edema volumes (%) of mice injected i.c.v. with Ctrl or VEGF-C at **c** 14 and **d** 35 days prior to ischemic surgery. **e**, **f** Exemplary T2-weighted MRI-series at 3 dpi of ischemic mice treated with either (**e**) VEGF-C or (**f**) Ctrl at − 14 dpi. *n* = 8–11; data points shown in graphs **c** and **d** represent individual mice. *P* values were calculated using two-tailed Student’s *t* test, *p* > 0.05

To study how VEGF-C affects the development of stroke, we injected adult mice with AAV-VEGF-C or AAV-Ctrl 14 or 35 days before tMCAo surgery (Fig. 2a, b). MRI-based quantification at 3 dpi did not reveal significant differences in the brain infarct or edema volumes between VEGF-C and Ctrl mice subjected to either treatment schedule (Fig. 2c–e). These findings indicated that i.c.v delivery of VEGF-C prior to stroke did not affect infarct expansion.

### VEGF-C Administration Reduces Ischemia-Induced Behavioral Deficits

**Fig. 3:**
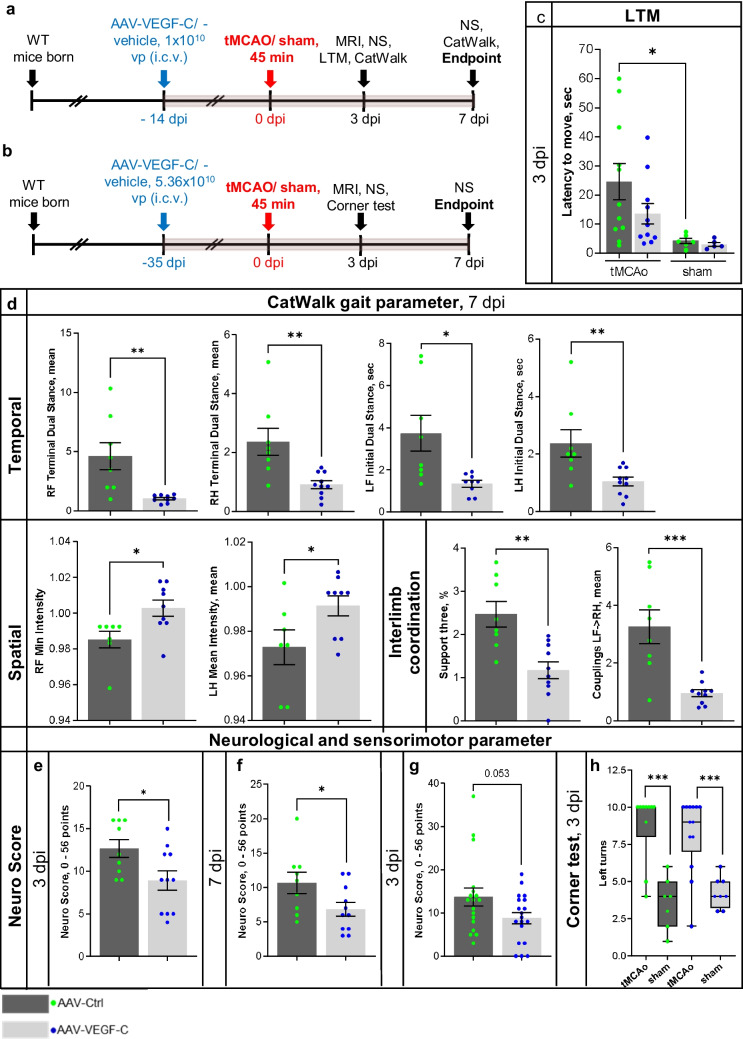
VEGF-C treatment improves gait and decreases neurological deterioration post-stroke. **a**, **b** Experimental outlines showing time points for AAV-administration, ischemic surgery, behavioral testing, and sample taking. **c** Latency to move test-results from day three post-surgery of mice the Ctrl or VEGF-C group. **d** Comparison of several temporal, spatial, and interlimb coordination parameters obtained from CatWalk gait analyses between ischemic mice at seven dpi of either Ctrl or VEGF-C treatment. **e**–**g** Neuro score comparison at **(e)** 3 or **(f)** 7 dpi of ischemic mice treated 35 days before surgery with Ctrl or as VEGF-C, as well as of mice treated (**g**) at 14 days prior to surgery. **h** Comparison of sensorimotor skills using the corner test in sham and ischemic mice treated with Ctrl or VEGF-C at 35 days prior to infarction. *n* = 6–19; data points shown in graphs **c**–**h** represent individual mice; statistical analyses were carried out using either two-tailed Student’s *t* test, or two-way ANOVA with Tukey’s post hoc test for multiple comparison, *p < 0.05, **p < 0.01, ***p < 0.005

Next, we wanted to determine if the pre-treatment with VEGF-C would affect locomotor activity, sensorimotor functions, or neurological outcome post-stroke. First, the locomotor activity of the mice was studied using the latency to move test at 3 dpi in mice treated with the AAVs 14 days before tMCAo (Fig. 3a). As expected, the tMCAo;Ctrl mice showed significantly increased latencies than the sham;Ctrl mice (*p* < 0.05, Fig. 3c). In contrast, tMCAo;VEGF-C mice did not show significantly increased latencies when compared with the sham;VEGF-C group (Fig. 3c). This suggested a mild VEGF-C-induced improvement in locomotor activity during the early phase after stroke at the time of testing.

A more detailed quantitative assessment in locomotor disturbances caused by ischemic stroke was done by applying CatWalk gait analyses [25, 29] at 7 dpi in mice injected with the AAVs 14 days before surgery. VEGF-C-treated mice showed significant improvement of locomotor disturbances post-stroke in comparison with the Ctrl-treated mice. VEGF-C-induced improvements included temporal, spatial, and interlimb coordination parameters (Fig. 3d). As temporal parameters, the terminal dual stance on the right front (RF) (*p* < 0.005) and hind (RH) paws (*p* < 0.004), and the initial dual stance of left front (LF) (*p* = 0.01) and hind (LH) paws (*p* < 0.01) were significantly reduced in the VEGF-C-treated mice (Fig. 3d). VEGF-C treatment resulted in improved spatial values of the minimal intensity of the RF paws (*p* = 0.02), as well as of the mean intensity of the LH paws in comparison with Ctrl treatment (*p* < 0.05; Fig. 3d). Furthermore, VEGF-C improved the interlimb coordination as shown by support three, a parameter that describes the number of paws used to support body weight during a step cycle [25] (*p* = 0.0016) and coupling (LF to RH, a temporal relationship between the placements of the LF and the RH paw within one step cycle; *p* < 0.001). These findings thus demonstrate that VEGF-C treatment improved locomotor gait abilities post-stroke.

### VEGF-C Treatment Improves General and Focal Neurological Outcomes Post-Stroke

In addition to gait impairment after ischemic stroke, we also accessed general and focal neurological deficits, which are eminent after focal ischemia, by neurological scoring according to Orsini and colleagues [31]. Mice injected with VEGF-C 35 days before tMCAo surgery (tMCAo;VEGF-C; Fig. 3b) showed significant improvement in neurological outcome both at 3 and 7 dpi when compared with the tMCAo;Ctrl group (*p* < 0.05, Fig. 3e, f). Mice treated with VEGF-C 14 days prior tMCAo surgery (Fig. 3a) also showed a trend toward a better neurological outcome when compared with tMCAo;Ctrl (*p* = 0.0531, Fig. 3g).

Corner test, measuring sensorimotor functions, showed a significant disturbance after ischemia (*p* < 0.001). However, in this test, we did not find a significant difference between the groups treated with VEGF-C or Ctrl 14 days before surgery (Fig. 3h). Collectively, these results demonstrate that i.c.v delivery of VEGF-C before stroke significantly improves early locomotor activity, locomotor gait disturbances, as well as general and focal neurological outcome post-stroke, whereas it has no effect on tested sensorimotor functions.

### Dynamic Gd-Enhanced MRI Reveals Several Stroke- and VEGF-C-Induced Changes in Brain Fluid Flow

We next assessed whether VEGF-C induces changes in the CSF circulation and outflow by quantifying the signal intensity in Gd contrast agent-enhanced MRI. Twenty-four hours after ischemia, we injected 2 µl of Gd-contrast agent, diluted at 1:16, i.c.v. at an infusion rate of 0.5 µl/min into the mice treated with AAV-VEGF-C or AAV-Ctrl 14 days before surgery. The quantified areas included the ischemic infarct area (Additional file 1. Fig. S1 a.1), the perifocal area of the brain (Additional file 1. Fig. S1 a.2), and the parenchyma of the basal brain (Additional file 1. Fig. S1 a.3). In the infarcted and perifocal cortex, the Gd-signal decreased steadily over time in the sham mice (sham;VC and sham;Ctrl). However, in the basal brain parenchyma, Gd-signal initially increased in all groups (first 15 min scan time), with the highest signal intensity in the sham;Ctrl mice (*p* > 0.05). After the initial increase, signal intensities decreased in all groups below the starting values. Interestingly, the sham;VC and sham;Ctrl mice showed no differences.

**Fig. 4:**
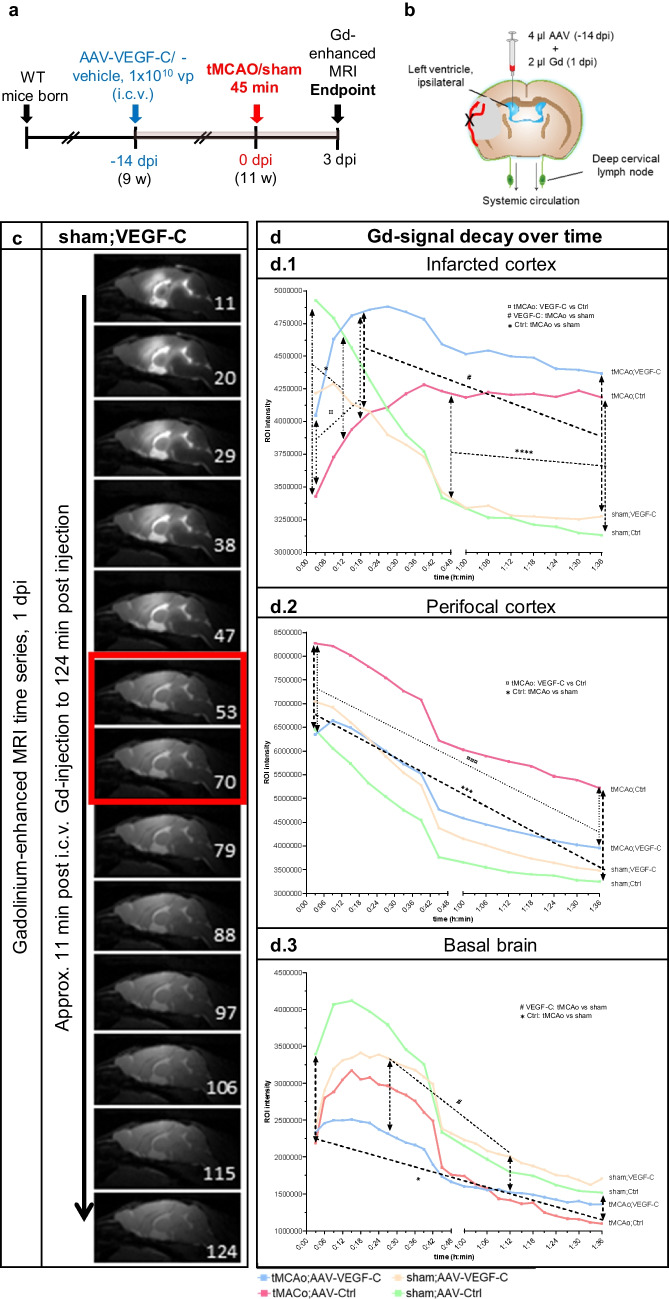
VEGF-C treatment increases Gd-signal in ischemic core and reduces it around the core. **a** Experimental outline showing time points for AAV-administration, ischemic surgery, and Gd-contrast agent enhanced MRI. **b** A schematic illustration of the AAV and Gd-contrast agent injection site at 14 days prior-tMCAo/sham-surgery, 24 h post-surgery respectively (including an indicative obstruction of the MCA (red) and an ischemic area in grey). **c** Dynamic contrast-enhanced MRI of a representative animal. T1-weighted MR images show the hyper intense Gd-signal penetrating the parenchyma after being injected into the lateral ventricle (2 µl Gd, 1:16 diluted, infusion rate of 0.5 µl/min; MRI scans taken every 9 min, red box indicates 17 min interruption due to the interposed anatomical (MGRE) scan). **d** Graphs show the Gd-signal intensity decline over time in areas relevant for the lymphatic outflow; (d.1) the area of the ischemic cortex, (d.2) the perifocal cortex adjacent, rostral the lateral ventricle, and (d.3) the basal brain parenchyma. *n* = 5–8; statistical analyses were carried out for two data points at once within 45 or 96 min (as indicated in the graphs d.1–3) by using the two-tailed Student’s *t* test

Ischemia had significant effect on Gd-signal. In the tMCAo;Ctrl mice, the initial Gd-signal in the infarcted cortex (Additional file 1. Fig. S1 a.1), was about 30% lower than in the sham mice (0–12 min, *p* < 0.001). The signal increased until 48 min, becoming 35% higher compared to sham;Ctrl mice and stayed significantly higher thereafter (*p* < 0.001; Fig. 4(d.1)), indicating delayed inflow and obstructed outflow in the infarct area. In the perifocal cortex (Additional file 1. Fig. S1 a.2), the Gd-signal was increased about 20% over the whole recording period but the shape of the curve remained unchanged vs sham mice (tMCAo;Ctrl vs. sham;Ctrl, 0–96 min; *p* < 0.001; Fig. 4(d.2)). In the basal brain, the Gd-signal was reduced throughout the recording period (tMCAo;Ctrl vs. sham;Ctrl mice, *p* < 0.05; Fig. 4(d.3)) without prominent differences in the flow dynamics. In tMCAo;VEGF-C and sham;VEGF-C mice, significant signal intensity differences were measured only during a limited time period (between 25 and 72 min recording time; *p* < 0.05; Fig. 4(d.3)) (Additional file 1. Fig. S2 depicts Fig. 4 d including SEM).

Unlike in the sham mice, VEGF-C treatment altered Gd-signal in ischemic mice. In the infarcted area, the initial Gd signal was 18% higher in tMCAo;VEGF-C vs. tMCAo;Ctrl mice and remained significantly higher for the first 18 min (*p* < 0.05; Fig. 4(d.1)), indicating altered flow dynamics within the ischemic tissue. In the perifocal area, VEGF-C treatment reduced the signal by 20% for the whole recording period when compared to Ctrl mice (tMCAo;VEGF-C *vs* tMCAo;Ctrl; *p* < 0.005; Fig. 4(d.2)), with a minor signal intensity difference compared to sham;Ctrl and sham;VEGF-C mice. In the basal brain parenchyma, VEGF-C treatment did not significantly change the Gd signal in comparison with Ctrl-treated mice (tMCAo;VEGF-C vs. tMCAo;Ctrl; *p* > 0.05; Fig. 4(d.3)).

**Fig. 5:**
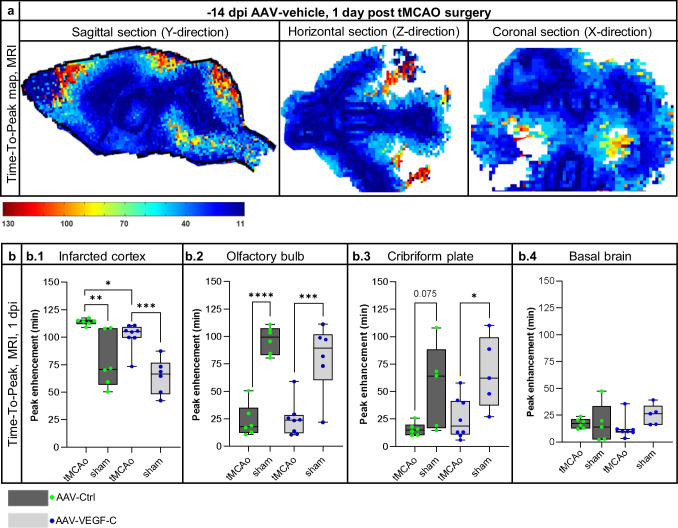
VEGF-C treated mice show significant alteration of the Gd-signal time-to-peak. **a** Representative time-to-peak map (in Y-, Z-, and X-dimensions), calculated from dynamic Gd-contrast enhanced image series at 1 dpi (color bar ranging from 11 to 130 min). **b**, **c** Graphs showing differences between tMCAo; Ctrl or tMCAo;VEGF-C, and/or sham; Ctrl/ sham; VEGF-C mice in the time needed for the peak enhancement (in min) within (b.1) the infarcted cortex, (b.2) the olfactory bulb, (b.3) the cribriform plate, and (b.4) the basal brain. *n* = 5–8; data points shown in graphs b.1–4 represent individual mice. Statistical analyses were carried out using the two-tailed Student’s *t* test or the one-way ANOVA followed by Sidak’s multiple comparisons post hoc test, *p ≤ 0.05, **p ≤ 0.01, ***p ≤ 0.005, ****p ≤ 0.001

In line with Gd-induced signal intensity changes, the voxel-wise quantification of the peak enhancement (Fig. 5a) showed significantly increased peaking time in the infarcted cortex compared to corresponding area in sham mice (tMCAo;Ctrl *vs* sham;Ctrl; *p* < 0.01; Fig. 5(b.1)). In tMCAo;Ctrl vs. sham;Ctrl mice, time needed to reach the maximum Gd-enhanced intensity was significantly reduced in the olfactory bulb (*p* < 0.005; Fig. 5(b.2)), and a similar trend was observed in the cribriform plate area (*p* = 0.075; Fig. 5(b.3)), whereas no effect was obtained in the basal brain parenchyma (Fig. 5(b.4)). VEGF-C treatment significantly reduced the peak enhancement time in the ischemic cortex compared to tMCAo;Ctrl mice (tMCAo;VEGF-C vs. tMCAo;Ctrl; *p* < 0.05; Fig. 5(b.1)). Although there was a significant stroke effect (tMCAo;VEGF-C *vs* sham;VEGF-C; *p* < 0.005; tMCAo;Ctrl vs. sham;Ctrl; *p* < 0.001), VEGF-C had no effect on peak enhancement times in the olfactory bulb.

### VEGF-C Treatment Reduces Astrogliosis and Increases Anti-Inflammatory Polarization of Microglia After Brain Ischemia

**Fig. 6:**
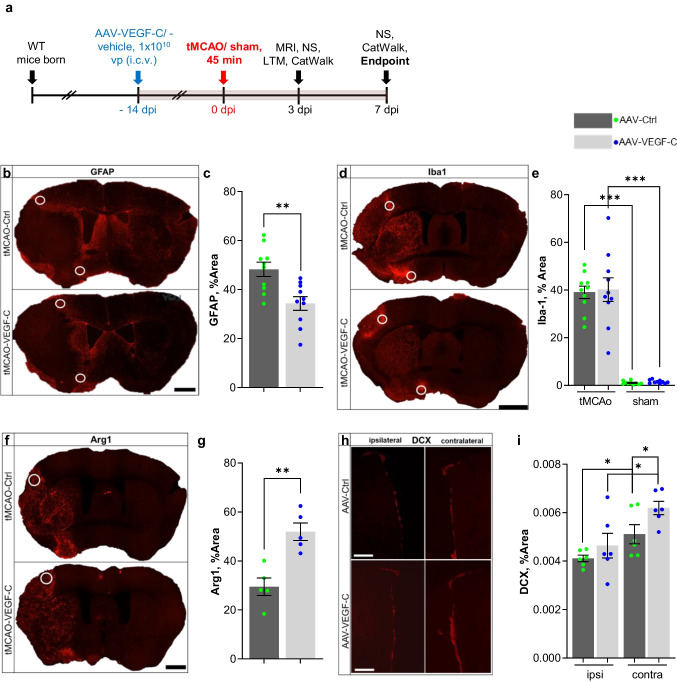
VEGF-C treatment leads to reduced GFAP- and enhanced Arg1-immunoreactivity in brain ischemia. **a** Experimental outline showing time points for AAV-administration, ischemic surgery, behavioral testing, and sample taking. **b** Anti-GFAP, **d** anti-Iba1, **f** anti-Arg1, and **h** anti-DCX stained brain sections from ischemic mice injected i.c.v. with Ctrl or VEGF-C; white circles indicate ROIs for analysis (**b**, **d**, **f**: scale bar = 1000 µm; **h**: scale bar = 100 µm). **c**, **g** Graphs show significant changes in (**c**) GFAP- or (**g**) Arg1-staining intensity between Ctrl- and VEGF-C-injected ischemic mice; (**e**) graph demonstrates differences in Iba1-staining intensities between Ctrl- and VEGF-C-treated ischemic and sham mice, and (**i**) shows DCX-staining intensities of the ipsi- and contralateral brain hemispheres between Ctrl- and VEGF-C-treated ischemic mice. GFAP *n* = 10; DCX *n* = 5/6; Arg1 *n* = 5/4; Iba1 *n* = 10. Data points shown in graphs **c**, **e**, **g**, and **i** represent individual mice. *P* values were calculated using a two-tailed Student’s *t* test; * *p* ≤ 0.05, **p ≤ 0.01,***p ≤ 0.001

Activation of astrocytes and microglia/macrophages in the early phase after stroke is known to be detrimental through their secretion of inflammatory mediators [36–40]. Therefore, we next investigated if VEGF-C treatment affects astrogliosis or microgliosis 3 days after ischemia in mice administered with AAVs 14 days before tMCAo (Fig. 6a). Astrocyte GFAP immunoreactivity was significantly reduced in the perifocal brain area of VEGF-C-treated mice compared to Ctrl mice (*p* = 0.0029; Fig. 6b, c). However, no difference was found between these groups in microglia as analyzed by Iba1 immunofluorescence (Fig. 6d, e). Interestingly, immunohistochemical staining of Arg1, a marker of alternatively activated microglia/macrophage phenotype [41], showed significantly more immunoreactivity in the lesion area in tMCAo;VEGF-C than tMCAo;Ctrl mice (*p* = 0.0022; Fig. 6f, g). AAV-VEGF-C has been demonstrated to induce VEFGR-3-mediated neurogenesis [42]. Injection of AAV-VEGF-C in the contralateral side to ischemia was able to induce significant neurogenesis when compared to the ctrl group as revealed by doublecortin staining. In addition, a significant increase in neurogenesis was observed on the contralateral side *vs* the ipsilateral side of tMCAo;VEGF-C group (*p* < 0.05, Fig. 6h, i). However, it was surprising to observe a similar upregulation of neurogenesis contralateral side of tMCAo;Ctrl groups.

### Dural Lymphatic Area Is Increased in VEGF-C-Treated Ischemic Mice

**Fig. 7:**
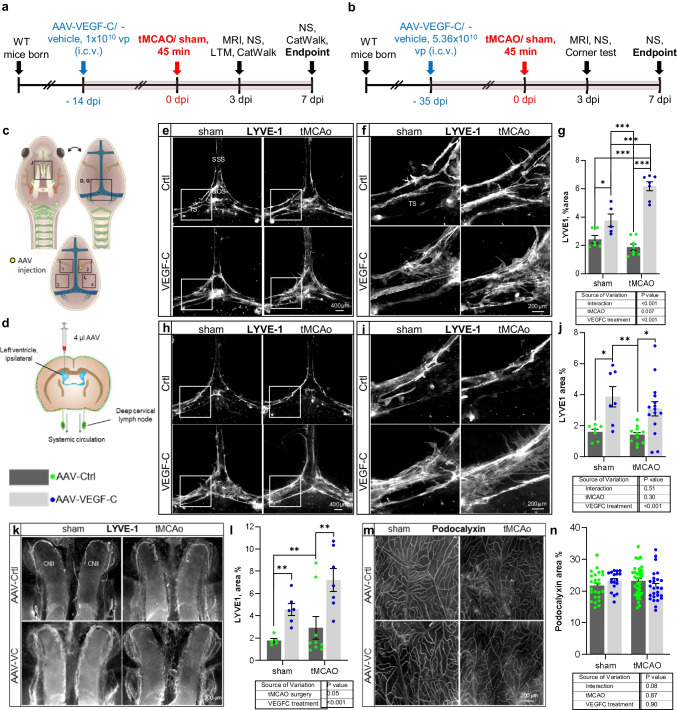
VEGF-C treatment increases dLV area in dorsal and basal skull. Comparison of Ctrl- and VEGF-C-treated sham and tMCAo mice. **a**, **b** Experimental outlines showing time points for AAV-administration, ischemic surgery, behavioral testing, and sample taking. **c** A schematic illustration of dLVs (green) attached to the ventral and dorsal sides of the cranium and spinal canal after removal of the brain and spinal cord indicating the quantified dLV and dBV areas. **d** A schematic illustration of the AAV injection site at 14 or 35 days prior-tMCAo/sham-surgery. **e**–**j** Comparison of LYVE-1 (white) staining in the dorsal dura mater at **e**–**g** − 14 dpi (*n* = 6, 5, 8, 7) and **h**–**j** − 35 dpi (*n* = 7, 7, 11, 15). **k**, **l** Comparison of LYVE-1 (white) staining in the basal dura mater at − 14 dpi (*n* = 5, 6, 9, 7). **m**, **n** Comparison of Podocalyxin (white) staining in the dorsal dura mater at − 14 dpi (*n* = 7, 5, 9, 8). The data shown are representatives of two independent experiments using littermate mice. Datapoints shown in graphs **g**, **j**, **l**, and **n** represent individual mice. *P* values were calculated using two-way ANOVA with Tukey’s post hoc test for multiple comparison. *p < 0.05; **p < 0.01; ***p < 0.005. **e **and **h**: scale bar = **400** µm; **f**, **i**, **k **and **m**: scale bar = 200 µm

As expected, mice injected with VEGF-C at 14 or 35 days prior tMCAo surgery showed significantly increased dLV area in dorsal and basal dura mater when compared with Ctrl-injected mice (treatment effect in 2-way ANOVA; *p* < 0.001 for both dorsal and basal dLVs; Fig. 7e–j). Although tMCAo alone did not increase the dLV area, it further increased the dLV area in VEGF-C-injected mice when the surgery was done 14 days, but not when surgery was done 35 days after VEGF-C injection (tMCAo surgery effect in 2-way ANOVA at − 14 dpi; *p* < 0.007 in dorsal; Fig. 7k, l). The blood vascular area in dura did not differ between the treatment groups (treatment effect in 2-way ANOVA at − 14 dpi; *p* = 0.9; Fig. 7 m, n).

## Discussion

In this study, we show that *Vefgc* gene delivery ameliorates stroke-induced locomotor and neurological deficits without altering the acute infarct size or brain edema when administered i.c.v. 14 or 35 days before the insult. This beneficial outcome was associated with increased dural lymphatic area and modulation of the brain fluid flow evident in the altered Gd-contrast agent outflow seen in MRI. Moreover, *Vefgc* gene delivery in the stroke mice triggered a phenotypic shift of microglia from pro-inflammatory to anti-inflammatory M2 state.

Stroke patients typically develop severe physical disabilities after their stroke. We used a transient MCA occlusion model that produces large, but somewhat variable-sized infarcts with accompanying behavioral and motor impairments, mimicking well the early outcome of stroke in patients. Importantly, administration of VEGF-C ameliorated neurological deficits at 3 and 7 dpi, indicating that increased expression of VEGF-C in brain improves functional recovery post-stroke. Likewise, VEGF-C treatment prevented the impairment in the latency to move test (LTM) of stroke mice, implying that the VEGF-C treatment also improved alertness and spontaneous activity of stroke mice although it did not decrease lesion size or brain edema. These observations are in line with previous studies showing that lesion size and LTM outcome do not necessarily correlate in mice [43] or functional recovery in humans. In addition, we were unable to detect sensorimotor improvement in VEGF-C treated mice at 3 dpi and our result is in agreement with a recently published paper where AAV-VEGF-C treated mice showed no improvement in sensorimotor function after stroke compared to control mice [44]. We furthermore used the CatWalk gait analysis system, which has been widely used to detect precise impairment of overall gait pattern and impairments of individual paws in transient ischemia models [45]. The VEGF-C-treated mice had improved spatial, interlimb coordination, and temporal gait locomotor abilities, which are the major functional deficits typically detected in the subacute post-stroke phase [25, 30].

Post-stroke inflammation depends on both the balance of pro- and anti-inflammatory processes [3]. The increase of anti-inflammatory M2a-like microglial cells has been shown to correlate with improved behavioral outcomes after stroke [25, 46, 47]. We found that *Vefgc* gene delivery increased the immunoreactivity of M2a-like microglia and M2a-macrophages after brain ischemia without altering the total amount of microglia or macrophages, detected in the perifocal area by Arg1 and Iba1-immunolabelling, respectively.

In the early phase after stroke, astrocytes secrete inflammatory mediators and free radicals and interfere with neuronal repair and synapse formation throughout scar formation [48]. Modulation and dampening of astroglial activation and proliferation is thought to promote neuronal plasticity and to improve functional outcome [49]. In our study, administration of VEGF-C significantly reduced astrocytic immunoreactivity in the peri-ischemic area of VEGF-C-treated mice compared to Ctrl mice. Taken together, immunomodulation of both microglia and astrocytes by VEGF-C likely contribute to functional recovery after stroke. Moreover, the VEGF-C treated mice showed an increased neurogenesis vs. control mice in the contralateral hemisphere where AAV-VEGF-C was injected. In fact, previous studies have already demonstrated that VEGF-C via its receptor VEGFR3 can activate neural stem cells and promote neurogenesis [50–52]. Although the increased neurogenesis did not reach statistical significance in the ipsilateral hemisphere, it was proposed to improve stroke outcome [19, 42].

We detected increased areas of dorsal and basal dural lymphatic vessels in AAV-VEGF-C treated mice when compared to control mice as previously described [8]. In addition, we observed ischemia-induced increase of dLV areas in AAV-VEGF-C treated mice 14 days but not 35 days prior to surgery. However, an increase in area does not necessarily correlate to functionality and at earlier time points lymphatic vessels are not fully matured. The observation in behavioral changes 14 days after AAV-VEGF-C injection might be due to the acute effect of VEGF-C.

Previous studies have shown that cerebral ischemia results in reduced CSF outflow, potentially hindering the clearance of toxic substances and molecular waste products in and around the ischemic tissue [4, 53]. Our analysis also revealed disruption of fluid flow from the brain after cerebral ischemia, as evidenced by the increased intensity of the Gd-contrast agents in the ischemic core approximately 30 min after ischemia. The fast influx of Gd into the ischemic area, as seen here by Gd-signal intensities, could possibly be explained by ischemia-induced CSF-influx caused by spreading depression (SD), as investigated by Mestre et al. 2020 [54]. In addition, Mestre and colleagues connected the enhanced fluid influx post-stroke, during SD, respectively, with a disruption of the physiological functions of AQP4 after stroke. The subtle increase in *vegfc* gene expression in cortical brain tissues after i.c.v. injection of the AAV-VEGF-C vector promoted growth of dLVs in both dorsal and basal parts of the skull. In the ischemic core area, Gd-signals were comparable between the AAV-VEGF-C injected mice and control mice. Interestingly, we observed that the VEGF-C treated ischemic mice exhibited a faster and stronger Gd-signal accumulation within the ischemic core area during the early phase after stroke as revealed by imaging. In contrast, we observed a lower Gd-signal intensity in the peri-ischemic area. Fluid outflow via the cribriform plate was also increased by VEGF-C treatment. Taken together, it seems reasonable to assume that VEGF-C promotes rapid drainage of fluid from perifocal areas to ischemic core during the early phase after stroke, hence behavioral recovery after stroke.

The role of AQP4 water channels in stroke recovery is controversial. AQP4 has been proposed to be important for the paravascular (glymphatic) solute flow [55], and indirect evidence indicates that after brain ischemia, AQP4 may contribute to early brain edema via CSF influx along perivascular spaces [56]. AQP4 has been suggested to regulate glymphatic flow, which could be disrupted during ischemia, leading to increased edema [57, 58]. We did not observe the change in mRNA levels of *Aqp4* between AAV-VEGF-C and control that did not undergo ischemia. In addition, we also did not observe a change in edema after ischemia between AAV-VEGF-C treated and ctrl mice. It can be speculated that VEGF-C might not have any beneficial effect on post-stroke behavioral improvement. However, further analysis of protein by either Western blot or IHC is required to confirm the acute modulation of VEGF-C in response to ischemic stroke and is one limiting factor in this study.

The tMCAo model was chosen as it is known to cause a relatively high variability in infarct volumes which is comparable to the variability of infarct volumes in humans. However, it is worth noting that, despite the involvement of two different surgeons in performing the tMCAo surgeries and the use of mice from different vendors (Envigo [C57BL/6JOlaHsd] and Janvier [C57BL/6JRj]), the infarction and edema volumes were comparable throughout all experimental groups. This suggest that the results obtained were not influenced by mouse substrain variation, which could potentially affect the response to stroke, such as the severity of post-stroke infection severity [59] or differences in gut microbiome composition, known to influence mouse behavior and locomotion [60].

Modulation of inflammation and improvement in neurological outcome documented in this study are in line with previously published articles where either VEGF-C pre-treatment or the use of K14-vegfre-lg transgenic mice have highlighted the importance of VEGF-C in ischemic stroke [44, 61]. Notably, manipulation of lymphatic vessels using either pre-treatment or viral vectors is not a suitable options. In addition, delivering VEGF-C after ischemic stroke seems to worsen the brain infarct without inducing lymphatic changes [62]. The effect of VEGF-C in the outcome after ischemic stroke appears to be context dependent and therefore further studies highlighting the specific context, the timing of the treatment and route of administration are crucial before thinking of translating into clinical practice.

In conclusion, our finding demonstrates that VEGF-C treatment leads to an improved neurological and behavioral outcome after stroke. This is further supported by a phenotypic shift toward anti-inflammatory glial cell types in the ischemic and peri-ischemic brain areas after stroke. Importantly, enhanced outflow of brain fluid and solutes towards the lymphatic system supports our hypothesis that VEGF-C-induced enhancement of the dLVs improves the stroke outcome.

## Supplementary Information

Below is the link to the electronic supplementary material.

Supplementary file1 (DOCX 1197 KB) Fig. S1. Representative anatomical MGRE-MRI images indicating the location of the ROIs. (a) Red circles demonstrate representative ROIs in the ipsilateral (a.1) ischemic brain parenchyma, (a.2) rostral perifocal brain parenchyma, and (a.3) the basal brain parenchyma; the green circle demonstrates a representative ROI in the contralateral basal brain parenchyma. in which the Gd-intensity enhancement/ decay over time was measured. Results are described in Fig. 4. (b) Shows two representative ROIs to detect the absolute value needed for the Gd-signal to peak, measured as min. Results are displayed in Fig. 5. MRI planes of x-,y-, and z- axes are displayed. Fig. S2. VEGF-C treatment causes significant alteration of the Gd-signal decay in ischemic brain. Graphs show the Gd-signal intensity decay over time including the standard error of the mean in areas relevant for the lymphatic outflow (a) area of the infarcted cortex, (b) the perifocal cortex adjacent to the lateral ventricle, (c) and the basal brain. n = 5-8; statistical analyses were carried out for one to two data points at once within 96 min (as indicated in the graph) by using the two-tailed Student`s t-test; p < 0.05.

## Data Availability

No datasets were generated or analyzed during the current study.

## Abbreviations

dpi: Days prior ischemia
A/P: Anterior/posterior
AAV: Adeno-associated virus
AQP4: Aquaporin4
Arg1: Arginase-1
CB: Cerebellum
CN II: Cranial nerve II/optic nerve
COS: Confluence of the sinuses
Ctrl: AAV without payload
Ctx: Cortex
D/V: Dorsal/ventral
dBV: Dural blood vessel
dcLN: Deep-cervical lymph node
DCX: Doublecortin
dLV: Dural lymphatic vessel
dpi: Days post ischemia
FISP: Fast imaging with steady-state precession
Gd: Gadolinium (contrast agent)
GFAP: Glial fibrillary acid protein
HC: Hippocampus
i.c.v.: Intracerebroventricular
Iba1: Ionized calcium-binding adapter molecule-1
LDF: Laser Doppler flowmetry
MRI: Magnetic resonance imaging
LF: Left front
LH: Left hind
LTM: Latency to move test
LYVE1: Lymphatic vessel endothelial hyaluronan receptor 1
M/L: Medial/lateral
MCA: Middle cerebral artery
MMA: Middle meningeal artery
OB: Olfactory bulb
PBS: Phosphate-buffered saline
PBS-TX: 0.3% Triton X-100 in PBS
PFA: Paraformaldehyde
PPA: Pterygopalatine artery
RF: Right front
RH: Right hind
ROI: Region of interest
RT: Room temperature
SSS: Superior sagittal sinus
ST: Cohort
Str: Striatum
TS: Transverse sinuses
VEGF-C: AAV vector encoding full-length mouse VEGF-C (AAV9-mVEGF-C-FL)
VEGF-C: Vascular endothelial growth factor
