# Supplementary material for: The Impact of VEGF-C-Induced Dural Lymphatic Vessel Growth on Ischemic Stroke Pathology
Source: Transl Stroke Res. 2024 Jun 1;16(3):781–99. doi: 10.1007/s12975-024-01262-9 (PMC12045824; doi:10.1007/s12975-024-01262-9)
Supplement: Supplementary file 1 — Supplementary file1 (DOCX 1197 KB) Fig. S1. Representative anatomical MGRE-MRI images indicating the location of the ROIs. (a) Red circles demonstrate representative ROIs in the ipsilateral (a.1) ischemic brain parenchyma, (a.2) rostral perifocal brain parenchyma, and (a.3) the basal brain parenchyma; the green circle demonstrates a representative ROI in the contralateral basal brain parenchyma. in which the Gd-intensity enhancement/ decay over time was measured. Results are described in Fig. 4. (b) Shows two representative ROIs to detect the absolute value needed for the Gd-signal to peak, measured as min. Results are displayed in Fig. 5. MRI planes of x-,y-, and z- axes are displayed. Fig. S2. VEGF-C treatment causes significant alteration of the Gd-signal decay in ischemic brain. Graphs show the Gd-signal intensity decay over time including the standard error of the mean in areas relevant for the lymphatic outflow (a) area of the infarcted cortex, (b) the perifocal cortex adjacent to the lateral ventricle, (c) and the basal brain. n = 5-8; statistical analyses were carried out for one to two data points at once within 96 min (as indicated in the graph) by using the two-tailed Student`s t-test; p < 0.05. [file 12975_2024_1262_MOESM1_ESM.docx]

Additional file 1


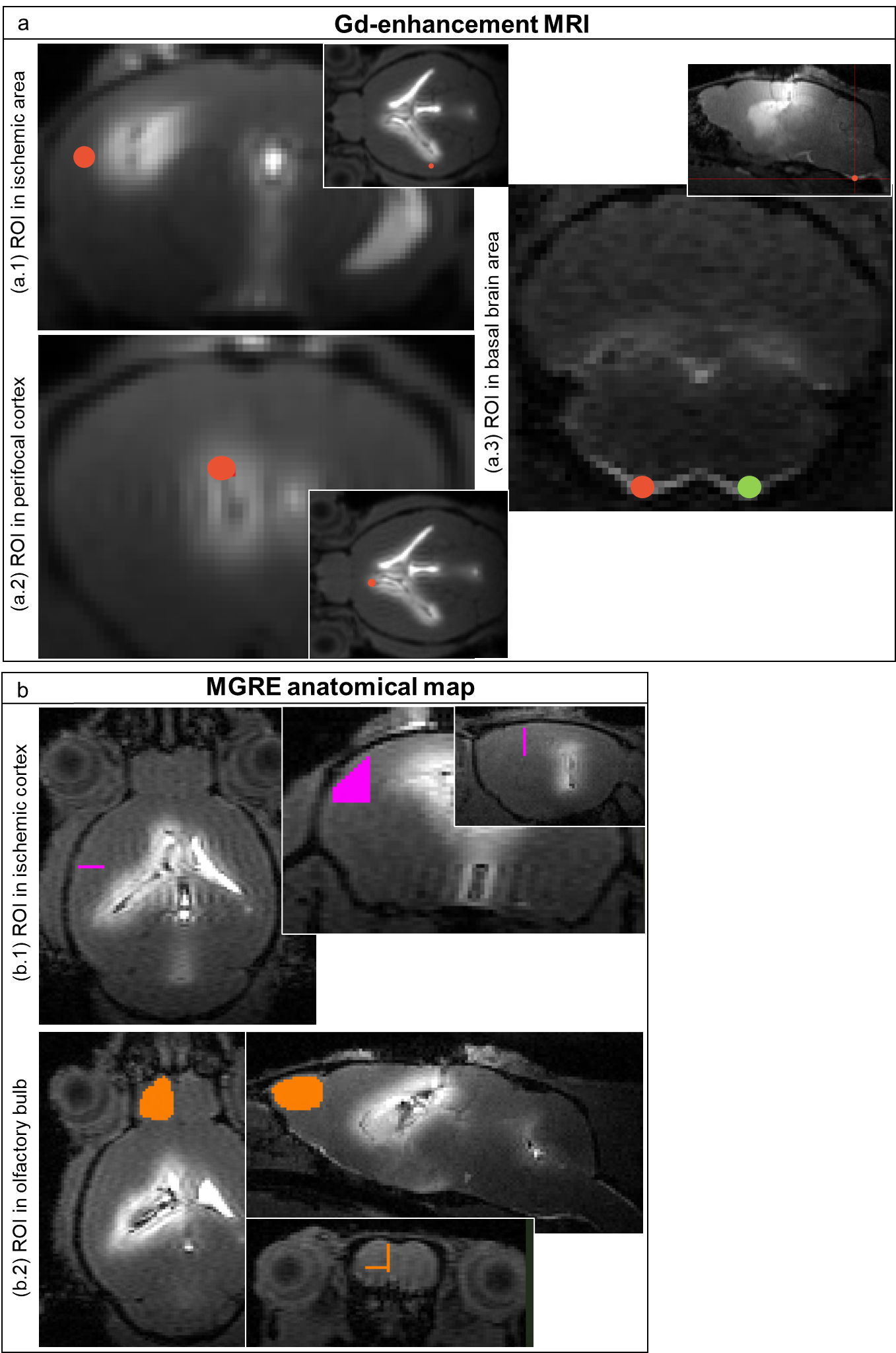


Fig: S1


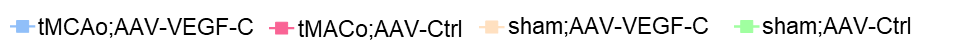


**Gd-signal decay over time**

Basal brain area

Infarcted cortex

Perifocal cortex

a

b

c

Fig. S2
